# Directing Traffic: Regulation of COPI Transport by Post-translational Modifications

**DOI:** 10.3389/fcell.2019.00190

**Published:** 2019-09-11

**Authors:** Peter M. Luo, Michael Boyce

**Affiliations:** Department of Biochemistry, Duke University School of Medicine, Durham, NC, United States

**Keywords:** COPI vesicle trafficking, coatomer, interphase, post-translational modifications, phosphorylation, ubiquitination, glycosylation, myristoylation

## Abstract

The coat protein complex I (COPI) is an essential, highly conserved pathway that traffics proteins and lipids between the endoplasmic reticulum (ER) and the Golgi. Many aspects of the COPI machinery are well understood at the structural, biochemical and genetic levels. However, we know much less about how cells dynamically modulate COPI trafficking in response to changing signals, metabolic state, stress or other stimuli. Recently, post-translational modifications (PTMs) have emerged as one common theme in the regulation of the COPI pathway. Here, we review a range of modifications and mechanisms that govern COPI activity in interphase cells and suggest potential future directions to address as-yet unanswered questions.

## Introduction

The coat protein complex I (COPI) mediates multiple lipid and protein trafficking paths between the endoplasmic reticulum (ER) and the Golgi. Mammalian COPI carries out this essential function through the combined action of seven core coatomer subunits: α-COP, β-COP, β′-COP, γ-COP, δ-COP, ε-COP, and ζ-COP (Popoff et al., 2011; Jackson, 2014; Rout and Field, 2017; Arakel and Schwappach, 2018; Bethune and Wieland, 2018). The genetic, biochemical, and structural details of the COPI pathway have been studied extensively. Here, we first provide a succinct overview of COPI mechanism and function, and we refer the interested reader to several excellent review articles for more comprehensive detail (Popoff et al., 2011; Jackson, 2014; Rout and Field, 2017; Arakel and Schwappach, 2018; Bethune and Wieland, 2018).

Briefly, COPI carrier formation is initiated when the small GTPase Arf1 exchanges GDP for GTP with the assistance of its guanine nucleotide exchange factor (GEF), GBF1 (Popoff et al., 2011; Jackson, 2014; Rout and Field, 2017; Arakel and Schwappach, 2018; Bethune and Wieland, 2018). Arf1-GTP inserts a myristoylated α-helix into the originating membrane (e.g., the *cis*-Golgi) and recruits the heptameric coatomer complex *en bloc* from cytoplasmic pools to the membrane (Popoff et al., 2011; Jackson, 2014; Rout and Field, 2017; Arakel and Schwappach, 2018; Bethune and Wieland, 2018). Stably bound COPI complexes assemble into an ordered lattice on the lipid bilayer, interacting with cargo through N-terminal domains of the α and β′ subunits, and promoting membrane curvature (Popoff et al., 2011; Jackson, 2014; Rout and Field, 2017; Arakel and Schwappach, 2018; Bethune and Wieland, 2018). Bilayer curvature triggers the recruitment of GTPase-activating proteins (GAPs) – ArfGAPs1-3 in mammals – which stimulate Arf1’s GTPase activity (Popoff et al., 2011; Jackson, 2014; Rout and Field, 2017; Arakel and Schwappach, 2018; Bethune and Wieland, 2018). GTP hydrolysis by Arf1 is required for cargo sorting, suggesting that COPI undergoes GTPase-driven conformational changes, though these steps are incompletely understood (Popoff et al., 2011; Jackson, 2014; Rout and Field, 2017; Arakel and Schwappach, 2018; Bethune and Wieland, 2018). Near the end of COPI assembly, extreme curvature promotes vesicle scission, also an Arf1-dependent event (Popoff et al., 2011; Jackson, 2014; Rout and Field, 2017; Arakel and Schwappach, 2018; Bethune and Wieland, 2018). Most COPI uncoats from the resulting, fully formed vesicle, likely coordinated by final rounds of GTP hydrolysis and the dissociation of Arf1 from the membrane (Popoff et al., 2011; Jackson, 2014; Rout and Field, 2017; Arakel and Schwappach, 2018; Bethune and Wieland, 2018). Finally, COPI vesicles dock to their destination membranes on the ER or Golgi. This step is aided by interactions among accessory proteins and lingering coatomer complexes on the vesicles, and by resident tethering and fusion components on the target organelle (Popoff et al., 2011; Jackson, 2014; Rout and Field, 2017; Arakel and Schwappach, 2018; Bethune and Wieland, 2018).

COPI is generally agreed to mediate retrograde protein and lipid trafficking within and from the Golgi to the ER, and, in animals, probably mediates anterograde trafficking from the ER-Golgi intermediate compartment (ERGIC) to the Golgi proper (Emr et al., 2009; Popoff et al., 2011; Jackson, 2014; Rout and Field, 2017; Arakel and Schwappach, 2018; Bethune and Wieland, 2018). The role of the COPI system in trafficking among the Golgi stacks is more controversial. One model invokes the forward movement of vesicles from *cis* to medial to *trans* Golgi cisternae, with COPI participating (Emr et al., 2009; Nakano and Luini, 2010; Glick and Luini, 2011; Papanikou and Glick, 2014). However, a newer and more broadly accepted model suggests that individual Golgi cisternae progressively acquire components and characteristics of *cis*, then medial, then *trans* Golgi character (Emr et al., 2009; Nakano and Luini, 2010; Glick and Luini, 2011; Papanikou and Glick, 2014). The role or requirement for COPI in this cisternal maturation process may be minor or nonexistent. Elements of both vesicular trafficking and cisternal maturation mechanisms could, in principle, co-occur within a single organism or cell type, and further research will be needed to clarify these ambiguities (Emr et al., 2009; Nakano and Luini, 2010; Glick and Luini, 2011; Papanikou and Glick, 2014). Nevertheless, COPI is a fundamental and essential pathway in the endomembrane system of all eukaryotic cells, regardless of its precise role in intra-Golgi trafficking.

The COPI pathway has been studied for decades through a range of elegant genetic, cellular, biochemical and biophysical studies, reviewed elsewhere (Popoff et al., 2011; Jackson, 2014; Rout and Field, 2017; Arakel and Schwappach, 2018; Bethune and Wieland, 2018). However, major aspects of COPI trafficking remain poorly understood. For example, little is known about how cells and tissues tune the activity of COPI trafficking in response to metabolic changes, physiological signals or stresses, such as the unfolded protein response or infection. In all of these contexts, COPI flux must adapt to the changing amount and type of client cargoes produced, but the nature of this rapid regulation is largely unclear. One key clue may come from related studies of Golgi dynamics during mitosis. The Golgi disperses in a regulated fashion during cell division for redistribution to the daughter cells, and numerous studies have dissected the mechanisms regulating this COPI-dependent process (Cancino and Luini, 2013; Huang and Wang, 2017). Several lines of evidence have defined a clear role for post-translational modifications (PTMs) in these cell cycle-dependent changes in Golgi structure (Cancino and Luini, 2013; Huang and Wang, 2017). Whether analogous mechanisms are used to tune COPI traffic in interphase cells is less well understood, but a variety of PTMs has been implicated in modulating interphase COPI activity, indicating the existence of robust and complex systems of regulation. Here, we focus specifically on major examples of interphase COPI regulation by PTMs and assess the future directions of this emerging area of cell biology.

## Phosphorylation

Protein phosphorylation has long been implicated in COPI regulation. For example, early studies found that inhibiting global dephosphorylation in hepatocytes disrupts the localization of β-COP and the architecture and function of the Golgi (Reaven et al., 1993), and demonstrated that β- and δ-COP purified from rat liver cytosol are themselves phosphorylated (Sheff et al., 1996). In the years since, several major kinase pathways were discovered to influence COPI function directly. Below we provide three examples of these links between phosphorylation and the COPI pathway. For a compendium of specific PTMs discussed throughout this review, see Table 1. For a continually updated and annotated database of all observed, COPI-relevant phosphosites and other PTMs, we direct the reader to the public PhosphoSitePlus resource^1^ (Hornbeck et al., 2015).

**TABLE 1:** Compendium of specific PTMs discussed in this review. Please see text for details.

**Substrate**	**PTM**	**Residue(s)**	**Modifying enzyme**	**Effect(s)**	**References**
**Coatomer components**					
α-COP	Phosphorylation	Unknown	PKA	Stimulates retrograde trafficking	Cancino et al., 2014
β-COP	Phosphorylation	Unknown	Unknown	Unknown	Sheff et al., 1996
γ1-COP	O-GlcNAc	Thr132, Ser134, Thr135, Thr552, Ser554	OGT	Unknown	Cox et al., 2018
δ-COP	Phosphorylation	Unknown	PKA	Stimulates retrograde trafficking	Cancino et al., 2014
ε-COP	Phosphorylation	Unknown	PKA	Stimulates retrograde trafficking	Cancino et al., 2014
ε-COP	Ubiquitination	Unknown	PIRH2	Proteasome-mediated degradation	Maruyama et al., 2008
ζ-COP	Phosphorylation	Unknown	PKA	Stimulates retrograde trafficking	Cancino et al., 2014
**COPI-related proteins and cargoes**					
aPKCI/l	Phosphorylation	Unknown	Src	β-COP recruitment to ERGIC, retrograde trafficking	Tisdale and Artalejo, 2006
Arf1	Myristoylation	N-terminus (GIy2)	N-myristoyltransferase	Membrane binding	Franco et al., 1996; Kaczmarek et al., 2017
ArfGAP1	Phosphorylation	Unknown	CKI	Modulates membrane binding	Yu and Roth, 2002
ArfGAP1	Phosphorylation	Unknown	LRRK2	Neurite shortening	Stafa et al., 2012
ASAP1	Phosphorylation	Tyr312	Src	Recruitment to Golgi membrane	Brown et al., 1998; Tsai et al., 2018
GBF1	Phosphorylation	Thr1337	AMPK	Attenuating GBF1 membrane association and function in Golgi disassembly	Miyamoto et al., 2008; Mao et al., 2013
HAS2	Posphorylation	Thr110	AMPK (?)	Promotes ER to Golgi trafficking	Melero-Fernandez de Mera et al., 2018
HAS2	O-GIcNAc and phosphorylation	Ser221	OGT and unknown kinase	Regulates rate of anterograde Golgi trafficking	Melero-Fernandez de Mera et al., 2018
HAS2	Ubiquitination	Lys190	Unknown	Promotes enzyme activity, possibly transport from the Golgi	Melero-Fernandez de Mera et al., 2018
KCNK3	Phosphorylation	Ser373	PKA	Recruits 14-3-3, permits anterograde transport	Kilisch et al., 2016
KCNK3	Phosphorylation	Ser393	PKA	Recruits 14-3-3, inhibits coatomer binding, promotes anterograde trafficking	O’Kelly et al., 2002; O’Kelly and Goldstein, 2008
KDEL receptor	Phosphorylation	Ser209	PKA	Increases ArfGAP and COPI interaction	Cabrera et al., 2003
p115	Phosphorylation	Ser941	CKII	Proposed to influence COPI vesicle tethering/docking	Dirac-Svejstrup et al., 2000
p24δ5	*N*-glycosylation	Asn86	Oligosaccharyltransferase	Promotes ERD2 binding and COPI-dependent retrograde trafficking	Pastor-Cantizano et al., 2017
Rab1	Phosphorylation	Thr75	TAK1	Essential for Rab1 function, inhibits GDI protein interaction	Levin et al., 2016
Snc1	Ubiquitination	Lys63	Rcy1-Skp1	Promotes COPI-mediated plasma membrane-endosome recycling in yeast	Xu et al., 2017
**Pathogen-directed modifications**					
Arf1	Proteolysis	After Gly2	IpaJ (*Shigella*)	Removes myristoylation, inhibits membrane binding	Burnaevskiy et al., 2013; Burnaevskiy et al., 2015
GBF1	Phosphorylation	Thr1337	AMPK and IRGM (hepatitis C virus)	Prolongs Arf1 activation, disrupts COPI trafficking	Hansen et al., 2017
Rab1	Adenylylation	Tyr77	SidM/DrrA (*Legionella*)	Disrupts COPI and other trafficking pathways	Muller et al., 2010; Neunuebel et al., 2011; Tan and Luo, 2011; Muller et al., 2012; Chen et al., 2013; Luitz et al., 2016
Rab1	Phosphocholination	Ser76	AnkX/LegA8 (*Legionella*)	Inhibits Rab1, remodels membrane trafficking	Mukherjee et al., 2011; Tan et al., 2011; Goody et al., 2012; Allgood et al., 2017
Rab1	Glucosylation	Thr75	SetA (*Legionella*)	Inhibits GTPase activity and GDI protein interaction	Wang et al., 2018

**FIGURE 1:**
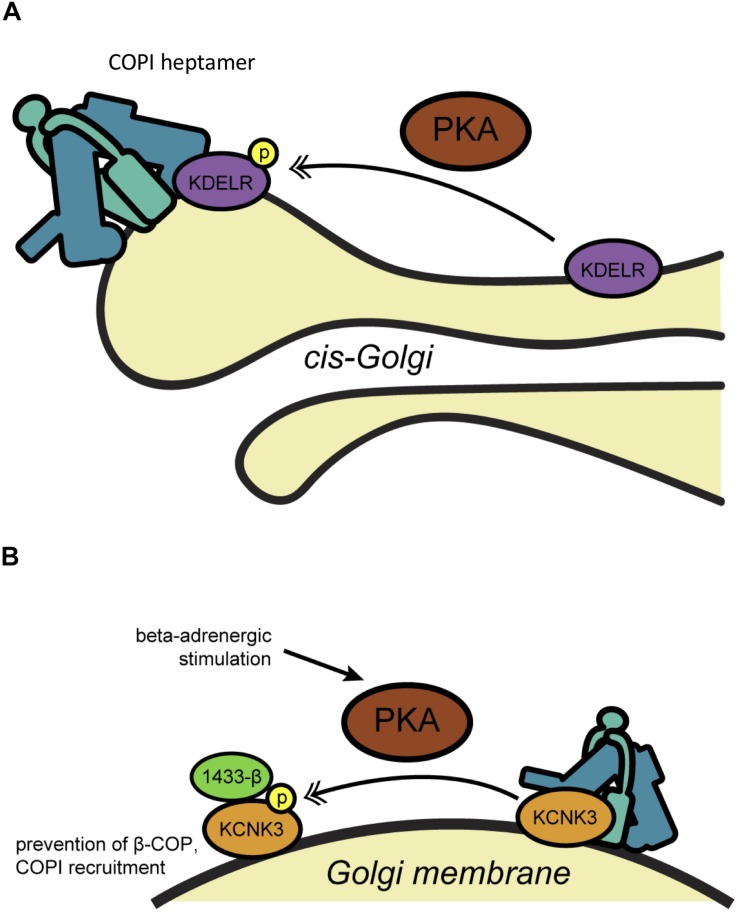
PKA-mediated phosphorylation regulates COPI trafficking. **(A)** Phosphorylation of KDELR by PKA on the Golgi membrane promotes its interaction with COPI (teal) and ArfGAP, regulating its recycling to the ER through COPI-dependent trafficking. **(B)** Phosphorylation of the potassium channel KCNK3 by PKA promotes 14-3-3β binding, displacing β-COP at the Golgi membrane and blocking retrieval to the ER allowing anterograde trafficking of the channel to the cell membrane. Single arrows indicate functional interaction. Double-headed arrows indicate translocation. COP, coat protein complex I; KCNK3, potassium channel subfamily K member 3; KDELR, KDEL receptor; P, phosphorylation; PKA, protein kinase A.

Cyclic AMP (cAMP)-dependent protein kinase A (PKA) is one of the best-documented regulators of interphase COPI trafficking (Figure 1). Early studies by the Velasco lab revealed that a small molecule inhibitor of PKA retarded the trafficking of the vesicular stomatitis virus G glycoprotein (VSVG) through the Golgi (Muniz et al., 1996). Conversely, PKA activators, such as forskolin, which stimulates cAMP synthesis, accelerated VSVG transport and altered Golgi cisternal structure, suggesting a possible impact on COPI (Muniz et al., 1996). Using *in vitro* reconstitution assays, the same group demonstrated that PKA or cAMP increased the binding of Arf1 to salt-washed Golgi membranes, whereas PKA inhibition or depletion, or dephosphorylation of Golgi membranes, had the opposite effect (Martin et al., 2000). The authors also showed that increases in cAMP levels in live cells triggered the redistribution of Arf1 from the cytoplasm to the Golgi, consistent with a regulatory role for PKA in COPI initiation (Martin et al., 2000). Subsequent work has supported this model, demonstrating that Golgi-associated PKA is rapidly activated by cAMP, inducing changes in the morphology of early Golgi stacks and accelerating trafficking (Mavillard et al., 2010). These results pointed to a possible role for Golgi-localized PKA in regulating the COPI pathway in response to extracellular signals.

The relevant PKA substrates and mechanism(s) of action in the COPI pathway remain incompletely understood, but current evidence points to several candidates. In an early clue that PKA signaling may influence membrane trafficking through Arf proteins in particular, the Vaughan lab showed that PKA binds and phosphorylates ArfGEF1 and -2, and both GEFs translocate from the cytosol to endomembranes upon forskolin-mediated cAMP upregulation (Li et al., 2003; Kuroda et al., 2007). Indeed, genetic knockdown of the PDE3A phosphodiesterase, which terminates cAMP signaling, decreased PKA-dependent ArfGEF membrane association and Arf1-GTP (Puxeddu et al., 2009). These results suggested a direct functional link between the PKA and Arf pathways, but the implications for COPI in particular remained uncertain, since ArfGEF1 and -2 are not major regulators of COPI trafficking. In subsequent work, both cytosolic and recombinant-purified PKA was also shown to phosphorylate the KDEL receptor (KDELR) itself at Ser209, promoting its interaction with COPI coatomer and ArfGAP (Cabrera et al., 2003) (Figure 1A). Although coatomer and ArfGAP1 can bind directly to unphosphorylated KDELR *in vitro* (Yang et al., 2002), PKA phosphorylation at Ser209 may play a regulatory role *in vivo*, as PKA inhibition prevented COPI-mediated retrograde transport of wild type, but not phosphomimetic Ser209Asp mutant, KDELR (Cabrera et al., 2003). In later work, anterograde ER-to-Golgi traffic was shown to trigger the G_*s*_ subunit of the heterotrimeric G protein complex and adenylyl cyclase, leading to the phosphorylation of α-, δ-, ε-, and ζ-COP and actin cytoskeletal regulators by PKA, ultimately stimulating retrograde trafficking to return KDELR and other COPI cargoes to the ER (Cancino et al., 2014). Taken together, these studies suggest a key role for PKA in balancing the anterograde and retrograde (i.e., COPI-dependent) pathways of Golgi trafficking to maintain organellar homeostasis. On the other hand, PKA is reported to have disparate effects on COPI trafficking, from promoting Arf1 membrane binding and transport (Martin et al., 2000; Cabrera et al., 2003; Mavillard et al., 2010; Cancino et al., 2014) to decreasing ArfGEF activity and Arf1-GTP levels (Li et al., 2003; Kuroda et al., 2007). It may be that spatially and temporally restricted activation (e.g., by cAMP and/or enzyme localization) allows PKA to exert divergent effects on trafficking in different contexts.

**FIGURE 2:**
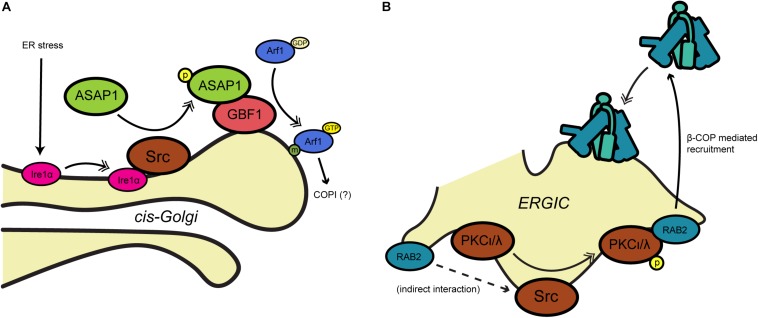
Src-mediated phosphorylation influences COPI trafficking. **(A)** During ER stress, Src associates with Ire1 and phosphorylates the ArfGAP ASAP1, leading to recruitment of Arf1-GTP to the Golgi membrane. **(B)** At the ERGIC, Src phosphorylates PKCι/λ, which is required by Rab2 to recruit β-COP for retrograde trafficking. Single arrows indicate functional interaction. Double-headed arrows indicate translocation. Arf, adenosine diphosphate-ribosylation factor; ASAP1, ArfGAP containing SH3, ANK repeat and PH domains; ERGIC, ER-Golgi intermediate compartment; GAP, GTPase activating protein; GBF1, Golgi-specific brefeldin A-resistance GEF 1; GEF, guanine nucleotide exchange factor; Ire1α, inositol-requiring 1; PKC, protein kinase C; Rab2, small GTPase; Src, non-receptor tyrosine kinase (name derived from “sarcoma”).

The non-receptor tyrosine kinase Src has also been implicated in COPI trafficking in several contexts. Early work by Brown et al. (1998) found that the ArfGAP ASAP1 interacts biochemically with the SH3 domain of Src and is tyrosine-phosphorylated, suggesting that ASAP1 might link Src signaling to membrane trafficking (Figure 2A). Although ASAP1 is thought to function primarily in regulating cytoskeletal rearrangements at the plasma membrane, recent work suggests that the Src/ASAP1 axis may impact on COPI as well. The Lee lab demonstrated that ER stress causes the integral ER membrane protein and stress sensor Ire1α to bind Src, promoting ASAP1 phosphorylation and recruitment to the Golgi membrane (Tsai et al., 2018) (Figure 2A). Relocalized ASAP1 bound to GBF1, increasing its GEF activity to facilitate ER stress-induced Arf1-GTP recruitment to the *cis*-Golgi (Tsai et al., 2018) (Figure 2A). These events likely impact on COPI, because ER stress-induced Src signaling and ASAP1 phosphorylation led to KDELR1 dispersion from the Golgi and suppression of retrograde trafficking (Tsai et al., 2018). In other work supporting the Src/COPI connection, Bard and coworkers observed collapsed Golgi stacks and distended cisternae in cells lacking the three ubiquitous Src-like kinases Src, Yes and Fyn (Bard et al., 2003). Expression of active Src induced KDELR dispersion from the Golgi, whereas transport of *Pseudomonas* exotoxin, a COPI-dependent KDELR client, was accelerated by chemical or genetic Src inhibition (Bard et al., 2003). Src also functionally interacts with the Rab family of GTPases, themselves important regulators of membrane trafficking (Hutagalung and Novick, 2011). Tisdale and Artalejo (2006) demonstrated that Rab2, which is required for transport between the ER and Golgi, promotes Src membrane association and its phosphorylation of the atypical kinase aPKCι/λ (see also below) on the ERGIC, leading to β-COP recruitment and retrograde trafficking (Figure 2B).

Other studies revealed that Src may also play a role analogous to PKA in regulating Golgi homeostasis by balancing forward and reverse transport. The Luini lab demonstrated that the anterograde trafficking of ER chaperones to the Golgi initiates a KDELR-dependent signaling cascade (Pulvirenti et al., 2008). Golgi-localized Src interacts with the KDELR directly, leading to Src activation and the upregulation of forward intra-Golgi transport through tyrosine phosphorylation (Pulvirenti et al., 2008). As with PKA and retrograde trafficking, then, Src activity may serve to balance anterograde transport to and within the Golgi to maintain organelle homeostasis.

More recently, the Bard lab reported a role for Src in growth factor-triggered redistribution of polypeptide *N*-acetylgalactosaminyltransferases (ppGalNAcTs), enzymes that initiate mucin-type O-glycosylation, from the Golgi to the ER (Gill et al., 2010). The authors reported that stimulation with epidermal growth factor or platelet-derived growth factor induces Src-driven redistribution of coatomer and ppGalNAcTs in an Arf1-dependent manner, implicating COPI trafficking in the reduction of mucin-type glycan biosynthesis through retrograde transport of glycosyltransferases (Gill et al., 2010; Chia et al., 2014). These results have been disputed, raising questions about the generality of the observations (Bard and Chia, 2017; Herbomel et al., 2017). Interestingly, however, the Bard lab has found evidence of O-glycosylation machinery relocalization to the ER in multiple human cancers, suggesting that activation of oncogenes like Src may dysregulate COPI or other trafficking pathways, resulting in aberrant glycoconjugates that might promote tumorigenesis (Gill et al., 2013; Nguyen et al., 2017). Testing this hypothesis will be an important goal of future work.

The protein kinase C (PKC) family is a final example of a well-documented COPI regulatory kinase. At the biochemical level, an early study showed that PKC promotes the binding of β-COP and Arf1 to Golgi membranes in a GTP-dependent manner, both in rodent cells and *in vitro* (De Matteis et al., 1993). Similarly, the Mochly-Rosen group showed that β′-COP interacts directly with PKCε *in vitro*, and the two proteins colocalize to the Golgi membrane of cardiomyocytes (Csukai et al., 1997). These results suggested that PKC may modulate COPI activity in response to upstream regulatory signals. Consistent with this notion, Westermann et al. (1996) showed that activation of membrane-bound PKC by phorbol 12-myristate 13-acetate increased the secretion of heparan sulfate proteoglycans from the *trans-*Golgi in a hepatocellular carcinoma cell line, though a specific role for COPI was not proven.

In another context, osmotically induced cell volume changes were shown to perturb COPI trafficking, as judged by an assay of brefeldin A-induced β-COP Golgi membrane dissociation (Lee and Linstedt, 1999). Interestingly, following several hours of osmotic stress, Golgi resident proteins returned to their proper location in a PKC-dependent (but protein synthesis-independent) manner, providing additional evidence for dynamic post-translational regulation of cargo trafficking by PKC (Lee and Linstedt, 1999).

Finally, as noted above, the Tisdale group identified a functional interaction between β-COP and PKC, focusing on the ι/λ isoform (Tisdale and Jackson, 1998; Tisdale, 2000) (Figure 2B). The authors showed that Rab2 requires PKCι/λ in order to promote the recruitment of β-COP to ERGIC membranes (Tisdale and Jackson, 1998; Tisdale, 2000) (Figure 2B). Perhaps surprisingly, β-COP recruitment did not require PKCι/λ kinase activity, whereas Rab2-mediated vesicle budding did (Tisdale, 2000). Though the mechanism remains to be fully elucidated, these results suggest that ERGIC-localized PKCι/λ may coordinate the Rab and coatomer families to regulate retrograde trafficking.

PKA, Src, and PKC represent perhaps the best-documented examples of interphase COPI regulation through phosphorylation. However, it is clear that more discoveries await us. Functional interactions have been reported between adenosine monophosphate-activated protein kinase (AMPK) and GBF1 (Miyamoto et al., 2008; Mao et al., 2013), casein kinase (CK) I and ArfGAP1 (Yu and Roth, 2002), and CKII and p115, a Golgi-resident COPI vesicle tethering protein (Dirac-Svejstrup et al., 2000) (Table 1). More recently, evidence has emerged that ArfGAP1 is regulated by the leucine-rich repeat kinase 2 (LRRK2), which is dysregulated in both inherited and sporadic forms of Parkinson’s disease (PD) (Stafa et al., 2012). The authors demonstrated that LRRK2 and ArfGAP1 interact *in vitro* and in brain tissue, and PD-associated mutations in LRRK2 alter this association (Stafa et al., 2012). Interestingly, ArfGAP1 promotes both the kinase and GTPase activities of LRRK2, and is phosphorylated directly by LRRK2 (Stafa et al., 2012). These effects may be functionally important in PD, because silencing ArfGAP1 expression in primary cortical neurons rescued the neurite shortening phenotype caused by overexpression of a disease-associated mutant LRRK2, whereas co-expression of ArfGAP1 and LRRK2 synergistically promoted neurite shortening (Stafa et al., 2012). In future work, it will be important to determine the role (if any) for COPI trafficking in LRRK2/ArfGAP1 signaling in the nervous system.

These and other results suggest that COPI trafficking is subject to a wide range of combinatorial inputs from diverse phosphorylation cascades. Supporting this general notion, genetic screens based on high-content imaging indicate that many additional kinases and phosphatases regulate secretion in general and COPI in particular, calling for further investigation (Farhan et al., 2010; Chia et al., 2012). Similarly, sophisticated mass spectrometry (MS)-based phosphoproteome profiling studies have demonstrated intriguing changes in the phosphorylation of coatomer proteins in response to such stimuli as glucose or insulin signaling (Sacco et al., 2016) and circadian oscillation (Robles et al., 2017). The responsible kinases remain to be identified experimentally, but computational analyses of these MS datasets suggest that PKA, PKC and CKII may account for many of the observed phosphosite changes, perhaps including those on COPI proteins (Sacco et al., 2016). We anticipate that efforts combining classical genetics and biochemistry with state-of-the-art imaging and MS approaches will reveal fascinating new mechanisms and functions of COPI regulation by phosphorylation.

## O-GlcNAcylation

O-linked β-*N*-acetylglucosamine (O-GlcNAc) is an abundant, single-sugar modification of serines and threonines on nuclear and cytoplasmic proteins (Hart, 2014; Bond and Hanover, 2015; Yang and Qian, 2017). Like phosphorylation, O-GlcNAc can cycle on and off substrates rapidly, sometimes on the timescale of minutes, thanks to the action of dedicated enzymes that add (O-GlcNAc transferase, OGT) and remove (O-GlcNAcase, OGA) the PTM. O-GlcNAc signaling governs myriad cellular processes and is dysregulated in many diseases, including cancer, diabetes and neurodegeneration (Hart, 2014; Bond and Hanover, 2015; Yang and Qian, 2017). Recent evidence also indicates that O-GlcNAcylation influences COPI trafficking (Figure 2). In a first report, Deng et al. (2014) used a protein microarray assay to discover direct biochemical interactions between OGT and several components of Golgi trafficking pathways, including ε-COP and several Rabs. These results hinted at a potential role for O-GlcNAcylation in COPI trafficking, but the relevant OGT substrate(s) remained unclear. Subsequently, our lab used a quantitative glycoproteomics approach and MS site-mapping to discover that γ_1_-COP (one of two mammalian γ isoforms) is O-GlcNAc-modified on at least 11 residues (Cox et al., 2018) (Table 1). Brefeldin A treatment reduced the O-GlcNAcylation of endogenous γ_1_-COP in human cells, suggesting a potential connection between coatomer glycosylation and pathway regulation (Cox et al., 2018). The functional impact of γ_1_-COP O-GlcNAcylation remains to be determined. However, because O-GlcNAc is a nutrient-sensitive PTM with ubiquitous roles in metabolic signaling (Ong et al., 2018; Hart, 2019), it is tempting to speculate that O-GlcNAcylation of γ_1_-COP or other coatomer components regulates COPI activity based on metabolic state. In addition, phosphorylation and O-GlcNAc are both O-linked modifications and can compete for identical or nearby residues on substrate proteins, giving rise to complex functional interplay between these PTMs (Hart et al., 2011) (see also below). Of note, five of the O-GlcNAc sites we identified on γ_1_-COP are also reported phosphosites, suggesting that coatomer may be regulated by crosstalk between these two O-linked PTMs (Cox et al., 2018). Experiments to test this possibility are underway.

## Myristoylation

Perhaps the first evidence of PTMs in the COPI pathway came from the discovery that Arf1 is myristoylated at its N-terminus (Kahn et al., 1988) (Table 1). This form of acylation, previously known to occur on other mammalian and viral GTPases, proved to be essential for Arf1’s membrane trafficking function (Franco et al., 1996; Kaczmarek et al., 2017). Mechanistically, myristoylation is required for the ability of GTP-bound Arf1 to bind target membranes and insert its α-helix into the bilayer (Antonny et al., 1997; Goldberg, 1998; Mossessova et al., 1998; Liu et al., 2009, 2010). To date, nearly all studies on Arf1 myristoylation have focused on its role in constitutive – as opposed to regulated – COPI trafficking. It will be interesting to learn from future work whether Arf1 myristoylation serves as a control point in any physiological contexts (e.g., if cells adjust the stoichiometry of available Arf1 via regulation of its acylation or the proteolytic removal of its myristoylated N-terminus). Several reports on the manipulation of Arf1 myristoylation by bacterial pathogens (see below) demonstrate the plausibility of this notion.

## Ubiquitination

**FIGURE 3:**
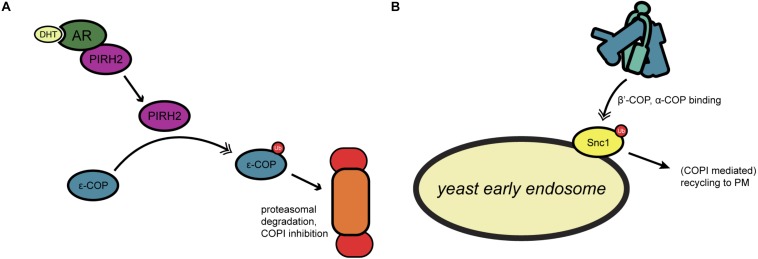
Regulation of COPI trafficking by ubiquitination. **(A)** In yeast, ubiquitination of the SNARE Snc1 allows for coatomer binding through β′- and α-COP, leading to recycling to the plasma membrane. **(B)** Androgen receptor signaling by dihydrotestosterone has been found to activate the ubiquitin ligase PIRH2, which targets ε-COP for proteasomal degradation. Single arrows indicate functional interaction. Double-headed arrows indicate translocation. AR, androgen receptor; DHT, dihydrotestosterone; PIRH2, p53-induced RING-H2 protein; PM, plasma membrane; Snc1, synaptobrevin homolog 1; Ub, ubiquitination.

Many COPI-relevant proteins are ubiquitinated in both yeast and mammals, including multiple coatomer components (Cohen et al., 2003; Hitchcock et al., 2003; Peng et al., 2003). However, little is known about the biochemical nature or functional impact that ubiquitination has on COPI activity. In one investigation of this question, Maruyama et al. (2008) examined mammalian ε-COP ubiquitination by PIRH2, an E3 ligase involved in androgen receptor (AR) signaling (Figure 3A). PIRH2 binds ε-COP and directly ubiquitinates it, promoting its proteasome-mediated degradation (Maruyama et al., 2008) (Figure 3A). In prostate cancer cells, dihydrotestosterone stimulation induced a PIRH2-AR interaction and concomitant ε-COP ubiquitination and degradation (Maruyama et al., 2008) (Figure 3A). In the same system, the authors demonstrated that overexpression of PIRH2 inhibits the secretion of prostate-specific antigen, suggesting a functional role for ubiquitination in linking hormone signaling to secretion (Maruyama et al., 2008).

In another example in yeast, the Kaminska lab built on prior proteomic studies (Hitchcock et al., 2003; Peng et al., 2003) to genetically dissect the role of ubiquitination in regulating actin remodeling and COPI trafficking (Jarmoszewicz et al., 2012). The authors demonstrated that a mutation in the *rsp5* ubiquitin ligase gene inhibits trafficking in loss-of-function genetic backgrounds for *ret1* or *sec28*, which encode α- and ε-COP, respectively (Jarmoszewicz et al., 2012). *Rsp5*/*ret1* double mutants exhibited reduced retrograde trafficking of the ER chaperone Kar2p and a hypersensitivity to neomycin, a drug that competes with coatomer for binding to dibasic motifs on COPI cargoes (Jarmoszewicz et al., 2012). These results indicate a functional impact of Rsp5p-mediated ubiquitination on Golgi-to-ER transport. Interestingly, the authors also identified a role for the actin cytoskeleton in these trafficking effects, as cells mutated in *arp2* (encoding an actin nucleating complex factor) or *sla1* (encoding a component of the PNA1 actin regulatory complex) also exhibit trafficking defects in a *rsp5* mutant background (Jarmoszewicz et al., 2012). It will be interesting to learn the direct ubiquitination targets of Rsp5p that mediate these effects on COPI trafficking and to determine whether its mammalian ortholog, NEDD4, has similar functions. NEDD4 is known to participate in other membrane trafficking events (Boase and Kumar, 2015), suggesting a potentially evolutionarily conserved impact on COPI as well.

## Trafficking Regulation Through Cargo PTMs

The above studies focused on COPI regulation through modification of the trafficking machinery itself. However, several lines of evidence indicate that PTMs on vectorially transported COPI cargoes can also influence their trafficking during interphase, providing another layer of regulatory control. Perhaps the best-studied example of this regulation is on potassium channel subfamily K (KCNK, also known as TASK-1) proteins (Figure 1B). In initial work, the Goldstein lab demonstrated that KCNK3 comprises two motifs that regulate its trafficking: a canonical N-terminal dibasic ER retrieval motif, which binds β-COP, and a C-terminal motif, which binds 14-3-3β in a phosphorylation-dependent manner (O’Kelly et al., 2002) (Figure 1B). The authors reported that β-COP binding and retrograde trafficking keep KCNK3 predominantly in the ER, whereas phosphorylation of the C-terminal motif on Ser393 by PKA recruits 14-3-3β, displaces β-COP and allows anterograde KCNK3 trafficking to the plasma membrane for active channel function (O’Kelly et al., 2002) (Figure 1B). Follow-up studies by the same group further clarified the mechanism of this regulation, demonstrating that 14-3-3 binding to KCNK3 promotes the subsequent binding of the p11 annexin in some tissues to promote forward transport of the channel (O’Kelly and Goldstein, 2008).

In complementary work, the Schwappach lab showed that phosphorylation of the C-terminal motif prevented β-COP binding to KCNK3 and the paralogous channel KCNK9 (also called TASK-3), even in the absence of 14-3-3 proteins (Kilisch et al., 2016). Interestingly, the authors identified another nearby PKA phosphorylation site, Ser373, on KCNK3 alone, which, when modified, inhibits COPI binding to permit anterograde transport (Kilisch et al., 2016) (Table 1). These results suggested that stimulus-induced PKA activity might modulate potassium signaling through regulated transport of ion channels. Supporting this model, the Schwappach lab reported that ATP-sensitive potassium channels, comprising Kir6.2 and SUR1 subunits, are retained in the Golgi in ventricular cardiomyocytes (Arakel et al., 2014). β-adrenergic stimulation, as would occur *in vivo* through sympathetic nervous system action, activates PKA, which phosphorylates the C-terminal domain of Kir6.2, displacing coatomer proteins and facilitating trafficking to the cell surface (Arakel et al., 2014) (Figure 1B). Beyond potassium channels, similar phosphorylation events have been reported to regulate the COPI association and forward trafficking of other cargoes, including major histocompatibility complex proteins and nicotinic acetylcholine receptors, suggesting that this mode of regulation may be widespread (O’Kelly et al., 2002; Khalil et al., 2005; Sand et al., 2014).

Cargo ubiquitination is also known to affect COPI transport. For example, Xu et al. (2017) showed that a subset of COPI coats localizes to the early endosome in yeast, and that the N-terminal WD40 propeller domains of both β′- and α-COP bind to Lys63-linked polyubiquitin on proteins, such as the v-SNARE Snc1 (Figure 3B). Deletion of the β′-COP propeller (but not mutation of the dibasic binding site alone) trapped Snc1 at the early endosome, whereas replacing the WD40 domain with unrelated ubiquitin-binding domains restored the recycling of Snc1 to the plasma membrane (Xu et al., 2017) (Figure 3B). These results suggest a role for yeast COPI in plasma membrane-endosome recycling that is mediated by recognition of polyubiquitin, rather than a dibasic motifs, on cargoes (Xu et al., 2017) (Figure 3B).

**FIGURE 4:**
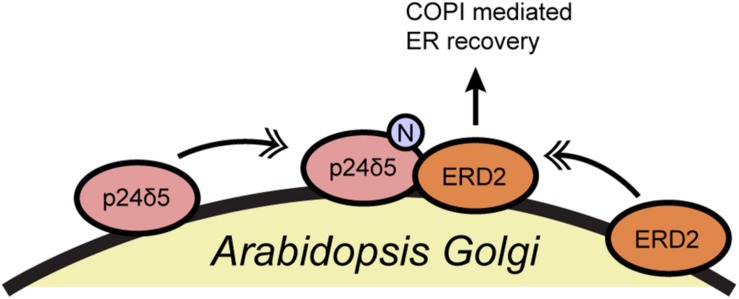
N-glycosylation of p24δ5 is required for ERD2 recycling in *Arabidopsis*. N-glycosylation of the COPI cargo p24δ5 promotes ERD2 binding and retrograde trafficking. Single arrows indicate functional interaction. Double-headed arrows indicate translocation. ERD2, ER lumen protein-retaining receptor 2; N, N-glycosylation.

In another example, the Aniento group reported that the N-glycosylation of a member of the p24 protein family affects the retrograde transport of the K/HDEL receptor ERD2 in plants (Pastor-Cantizano et al., 2017) (Figure 4). p24 proteins associate with COPI vesicles and likely participate in coatomer recruitment and cargo selection, particularly for glycosylphosphatidylinositol-anchored proteins, but they remain incompletely understood (Pastor-Cantizano et al., 2016). Aniento and coworkers demonstrated that N-glycosylation of *Arabidopsis* p24δ5 is required for its binding to ERD2 and for the COPI-dependent trafficking of ERD2 from the Golgi to the ER (Pastor-Cantizano et al., 2017) (Figure 4). p24δ5 glycosylation does not affect its binding to the coatomer itself, and so the precise biochemical mechanism of this regulation remains to be determined (Pastor-Cantizano et al., 2017). However, several fungal and mammalian p24 proteins are glycosylated as well, suggesting a possible evolutionarily conserved mode of retrograde trafficking regulation through glycosylation, an interesting topic for future work.

As these examples show, a range of cargo PTMs can influence COPI transport (Table 1), raising the possibility of combinatorial control of COPI trafficking through multiple PTMs on individual cargoes. A recent report on hyaluronan synthase 2 (HAS2) by the Deen group illustrates this possibility (Melero-Fernandez de Mera et al., 2018) (Table 1). The authors showed that phosphorylation of HAS2 at Thr110 is required for its transit from the ER to the Golgi and subsequently to the plasma membrane (Melero-Fernandez de Mera et al., 2018). In addition, HAS2 Ser221 is alternatively O-GlcNAcylated or phosphorylated, affecting HAS2 by controlling both its rate of anterograde trafficking through the Golgi and its rate of endocytosis from the plasma membrane to endolysosomes, where it is degraded (Melero-Fernandez de Mera et al., 2018). Finally, the authors show that HAS2 ubiquitination on Lys190 is essential for its activity and may play a role in its transport from the Golgi to the plasma membrane (Melero-Fernandez de Mera et al., 2018). At present, little is known about the upstream factors controlling HAS2 PTMs or whether they impinge directly on COPI transport and/or other trafficking pathways. Nevertheless, these results highlight the possibility that dynamic stimuli might signal through combinations of diverse PTMs to control cargo movement through COPI and other transport systems.

## Pathogen Subversion of Copi Trafficking Through PTMs

**FIGURE 5:**
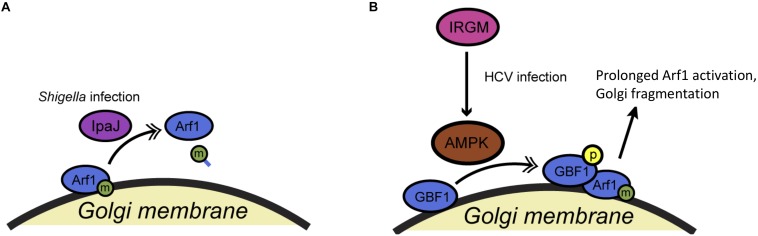
Pathogens manipulate COPI trafficking during infection. **(A)** The *Shigella flexneri* type III effector protein IpaJ cleaves the myristoylated N-terminus of Arf1 during infection, disrupting its membrane association and COPI trafficking. **(B)** During hepatitis C virus infection, phosphorylation of GBF1 is triggered by host IRGM and a kinase, likely AMPK, leading to prolonged Arf1 activation and disruption of normal trafficking. Single arrows indicate functional interaction. Double-headed arrows indicate translocation. AMPK, adenosine monophosphate-activated protein kinase; HCV, hepatitis C virus; IpaJ, invasion plasmid antigen J (*Shigella*); IRGM, immunity-related GTPase M.

Like many fundamental cell biological processes, COPI trafficking can be subverted by pathogens to promote their own survival and growth, and PTMs have been implicated in this phenomenon (Figure 5 and Table 1). For example, the Alto lab demonstrated that the IpaJ type III effector protein of *Shigella flexneri* is a cysteine protease specific for the myristoylated N-terminus of Arf proteins (Burnaevskiy et al., 2013) (Figure 5A). *Shigella* infection had been reported previously to disrupt Golgi morphology, but the mechanism remained uncertain. As the authors showed, IpaJ cleaves the peptide bond between the myristoylated-Gly2 and Arg3 residues of Arf1, effectively deacylating the protein and presumably directly causing the dramatic impact on COPI trafficking and Golgi homeostasis observed during *Shigella* infection (Burnaevskiy et al., 2013). Interestingly, IpaJ can cleave a range of N-myristoylated host proteins *in vitro* but exhibits remarkable specificity for Golgi GTPases in infected cells (Burnaevskiy et al., 2015). The specific advantages that *Shigella* derives from sabotaging COPI transport are not entirely clear, but these results raise the intriguing possibility that other pathogens – and perhaps even endogenous cellular machinery – may manipulate trafficking through Arf1 deacylation.

In a second, recent example, Hansen et al. (2017) demonstrated that the human immunity-related GTPase M (IRGM) influences Golgi trafficking during hepatitis C virus (HCV) infection (Figure 5B). The authors show that IRGM localizes to the Golgi and participates in HCV-directed Golgi fragmentation (Hansen et al., 2017). Specifically, HCV infection triggers the IRGM-mediated phosphorylation of GBF1, probably by AMPK (Hansen et al., 2017). This PTM likely prolongs Arf1 activation, disrupting the COPI pathway and fragmenting the Golgi to promote viral replication (Hansen et al., 2017). It will be interesting to dissect the mechanism and effects of HCV’s manipulation of IRGM during infection, and to determine the normal role that IRGM plays, if any, in the COPI pathway of healthy cells.

Rab1 is a small GTPase known to regulate several membrane trafficking pathways, including COPI (Hutagalung and Novick, 2011; Yang et al., 2016; Saraste and Marie, 2018). Recently, Rab1 has also emerged as a major target of several pathogen-directed PTMs, particularly by the bacterium *Legionella pneumophila*, the causative agent of Legionnaire’s disease (Machner and Chen, 2011; Goody and Itzen, 2013; Misch, 2016). *Legionella* induces the reversible adenylylation of Rab1, with the secreted bacterial effector proteins SidM/DrrA and SidD covalently adding and removing adenosine monophosphate (AMP) to Rab1, respectively, at different time-points during infection (Muller et al., 2010, 2012; Neunuebel et al., 2011; Tan and Luo, 2011; Chen et al., 2013; Luitz et al., 2016) (Table 1). Rab1 AMPylation locks it into its active (GTP-bound) conformation, presumably to manipulate host membrane transport pathways, though whether COPI trafficking or other processes regulated by Rab1 (e.g., endocytosis, autophagy) are the key targets for *Legionella* remains unclear (Derre and Isberg, 2004; Kagan et al., 2004; Joshi and Swanson, 2011; Mukherjee et al., 2011). Complicating the picture, the coopting of Rab1 by SidM/DrrA binding also occurs through PTM-independent mechanisms, including alteration of the Rab1 GTPase activity and blocking the association of Rab1 with host GDP-dissociation inhibitor (GDI) proteins (Machner and Isberg, 2006, 2007; Murata et al., 2006; Ingmundson et al., 2007). More work will be needed to understand the mechanisms and downstream functional effects of SidM/DrrA and SidD during *Legionella* infection.

Remarkably, Rab1 is subject to other PTMs by *Legionella* effectors as well. For example, the bacterial protein AnkX/LegA8 attaches a phosphocholine lipid moiety to Rab1, a PTM that can be removed by pathogen-encoded Lem3/lpg0696 (Mukherjee et al., 2011; Tan et al., 2011; Goody et al., 2012; Allgood et al., 2017) (Table 1). Phosphocholination inhibits Rab1 function and is required for membrane remodeling during *Legionella* infection (Mukherjee et al., 2011; Tan et al., 2011; Goody et al., 2012; Allgood et al., 2017). More recently, the *Legionella* effector SetA was shown to glucosylate Rab1, inhibiting its GTPase activity and its interaction with GDI proteins (Wang et al., 2018) (Table 1). Taken together, these studies demonstrate that *Legionella* invests significant resources in hijacking Rab1, suggesting an important role for the remodeling of endomembrane traffic during infection. It will be important to learn the particular pathways controlled by Rab1 that are most critical during *Legionella* pathogenesis, and whether these are differentially affected by discrete pathogen-directed PTMs (Mukherjee et al., 2011; Tan et al., 2011; Goody et al., 2012; Allgood et al., 2017; Wang et al., 2018).

Recently, research on pathogen/Rab1 interactions has facilitated separate discoveries on the native regulation of Rab1 by endogenous PTMs. Mammalian TGF-β-activated kinase 1 (TAK1), which regulates the AP-1 and NF-κB transcription pathways during innate immune signaling, was found to phosphorylate Rab1 at a site on its switch II region, near the hotspot modified by *Legionella* effectors (Levin et al., 2016) (Table 1). Phosphorylation of Rab1 by TAK1 disrupts its interaction with GDI proteins (but not GEFs or GAPs), and is essential for Rab1 function (Levin et al., 2016). Notably, *Legionella* infection reduces Rab1 phosphorylation by TAK1, suggesting that the pathogen may subvert normal cellular Rab1 PTMs as a way of interfering with TAK1-mediated innate immune signaling (Levin et al., 2016). This work provides an excellent example of how discoveries from the eukaryotic trafficking and microbial pathogenesis fields can illuminate each other.

## Conclusion

Partitioning proteins and lipids into specialized compartments is a signature feature of all eukaryotes. To create and maintain this organellar homeostasis, cells rely on a variety of essential and highly regulated trafficking pathways, including COPI. Since its discovery, great strides have been made in understanding the structure and function of COPI in constitutive vesicle formation and trafficking. Despite these achievements, however, we know relatively little about the dynamic regulation of COPI transport in interphase cells experiencing fluctuating signals, nutrients, developmental cues or stresses. PTMs clearly provide one – though not the only – broad mechanism that eukaryotes use to tune COPI activity. The studies reviewed here highlight the role that diverse PTMs play in interphase COPI trafficking. We anticipate that recent advances in high-content imaging, MS, genome engineering, structural biology and computational modeling will reveal new examples as well, eventually leading to an integrated model for how cells modulate COPI transport in real time. To this end, one important future goal will be to comprehensively site-map functionally important PTMs on coatomer proteins, Arfs and ArfGEFs, and integrate this information with improved structural data on the COPI coat, in order to gain biophysical insight into the mechanism of trafficking regulation by COPI PTMs. Knowledge from such efforts may also shed light on other, longstanding questions in the COPI field, such as why mammals encode multiple similar isoforms of some critical COPI components (e.g., γ- and ζ-COP, GBF1 isoforms) and how these may be differentially regulated by PTMs, how under-studied PTMs govern other components of the COPI pathway (e.g., ArfGAPs), how COPI transport influences the distribution of lipids and glycans, which PTMs are required in intact tissues and organisms to maintain COPI homeostasis, and how these processes might be dysregulated in disease. We expect that exciting new answers to these questions will be found in the coming years, with PTMs playing a central role.

## Author Contributions

PL and MB wrote the manuscript, performed revisions, and read and approved the submitted version.

## Conflict of Interest Statement

The authors declare that the research was conducted in the absence of any commercial or financial relationships that could be construed as a potential conflict of interest.

## Footnotes

1 https://www.phosphosite.org
